# Angiotensin type 2 receptor activation promotes browning of white adipose tissue and brown adipogenesis

**DOI:** 10.1038/sigtrans.2017.22

**Published:** 2017-06-23

**Authors:** Aung Than, Shaohai Xu, Ru Li, Melvin Khee-Shing Leow, Lei Sun, Peng Chen

**Affiliations:** 1 School of Chemical and Biomedical Engineering, Nanyang Technological University, Singapore, Singapore; 2 Duke-NUS Graduate Medical School, Singapore, Singapore; 3 Endocrine and Diabetes, Tan Tock Seng Hospital, Singapore, Singapore

## Abstract

Brown adipose tissue dissipates energy in the form of heat. Recent studies have shown that adult humans possess both classical brown and beige adipocytes (brown-like adipocytes in white adipose tissue, WAT), and stimulating brown and beige adipocyte formation can be a new avenue to treat obesity. Angiotensin II (AngII) is a peptide hormone that plays important roles in energy metabolism via its angiotensin type 1 or type 2 receptors (AT1R and AT2R). Adipose tissue is a major source of AngII and expresses both types of its receptors, implying the autocrine and paracrine role of AngII in regulating adipose functions and self-remodeling. Here, based on the *in vitro* studies on primary cultures of mouse white adipocytes, we report that, AT2R activation, either by AngII or AT2R agonist (C21), induces white adipocyte browning, by increasing PPARγ expression, at least in part, via ERK1/2, PI3kinase/Akt and AMPK signaling pathways. It is also found that AngII–AT2R enhances brown adipogenesis. In the *in vivo* studies on mice, administration of AT1R antagonist (ZD7155) or AT2R agonist (C21) leads to the increase of WAT browning, body temperature and serum adiponectin, as well as the decrease of WAT mass and the serum levels of TNFα, triglycerides and free fatty acids. In addition, AT2R-induced browning effect is also observed in human white adipocytes, as evidenced by the increased UCP1 expression and oxygen consumption. Finally, we provide evidence that AT2R plays important roles in hormone T3-induced white adipose browning. This study, for the first time, reveals the browning and brown adipogenic effects of AT2R and suggests a potential therapeutic target to combat obesity and related metabolic disorders.

## Introduction

The excess white adipose tissue (WAT) that characterizes obesity is a major risk factor for the development of many diseases, such as diabetes, coronary heart disease, hypertension, stoke and some types of cancers (for example, colorectal cancer).^1,2^ In contrast to the notorious WAT that stores energy as lipids, brown adipose tissue (BAT) dissipates energy directly as heat by uncoupling oxidative phosphorylation from adenosine triphosphate (ATP) production through the action of brown adipocyte-specific uncoupling protein 1 (UCP1).^3^ BAT also improves insulin sensitivity and regulates glucose homeostasis.^4^ Recent studies have shown that metabolically active BAT, which was thought to disappear after infancy, is actually present in adult humans.^5^ Although BAT is rare (present as small clusters in supraclavicular and paraspinal regions) in adult humans and even less in obese people, brown-like adipose cells (called beige cells) have been identified in certain WAT depots (for example, subcutaneous WAT).^6,7^ Emerging evidence suggests that beige adipocytes in WAT promote body energy consumption, and confer beneficial effects on obesity and insulin resistance.^6,8^ The development of beige cells in WAT (a process known as WAT browning) occurs in response to prolonged cold exposure (or β-adrenergic stimulation), and some hormones (for example, thyroid hormone T3, fibroblast growth factor 21).^8^ Thus, the recent discovery of inducible beige cells in WAT have inspired the possibility to treat obesity and related metabolic disorders through stimulating ameliorative self-remodeling of WAT.

WAT is not just a passive depot for lipid storage, but also the largest endocrine organ secreting a variety of signaling molecules called adipokines, which play critical roles in regulating energy metabolism in adipose tissue and whole body.^9,10^ Recent studies demonstrated that adipokines also modulate WAT browning. For examples, TNFα suppresses UCP1 expression in white adipocytes via extracellular signal-related kinase (ERK) activation^11^ whereas both apelin and adiponectin enhance WAT browning.^12,13^ Angiotensin II (AngII) is an adipokine well-known for its crucial roles in regulating energy homeostasis (for example, by influencing food intake, insulin secretion, glucose uptake), and its involvements with the development of obesity and type2 diabetes.^14^ AngII, the major bioactive component of the renin-angiotensin system (RAS), is produced from angiotensinogen by the actions of renin and angiotensin-converting enzyme (ACE), and acts through angiotensin type 1 and type 2 receptors (AT1R and AT2R). Studies have revealed that all components of RAS (including AT1R and AT2R) are present in adipose tissue,^15–17^ implying the involvement of local RAS in regulating adipose functions and self-remodeling. This hypothesis has been supported by a number of investigations on RAS regulations of adipocyte differentiation, lipid metabolism, as well as expression and release of adipokines.^15,17,18^ Most of AngII effects reported in adipocytes are negative and mediated by AT1R, such as, reduction of glucose uptake, induced oxidative stress, increased release of pro-inflammatory cytokines, and stimulation of adipocyte hypertrophy.^17,19^ In contrast, except for its roles in promoting adipocyte differentiation and insulin sensitivity via PPARγ activation,^20,21^ AT2R’s effects in adipocyte are much less elucidated. In particular, the implication of AT2R signaling on adipose browning is unknown.

It has been shown that chronic treatment with AT2R agonist (C21) reduces the size of hypertrophic adipocytes and adipose tissue mass in rodents fed with high-fat diet,^20,21^ suggesting the favorable effects of AT2R activation for obesity. Because the plasma level of AngII is elevated in obesity,^22^ we speculate that AT2R activation serves to counteract the often detrimental actions by AT1R. Here, we reveal the currently-elusive AT2R signaling implicated in WAT browning, brown adipogenesis, and thyroid hormone T3-induced white adipocyte browning.

## Materials and methods

### Primary pre-adipocyte culture and differentiation

The primary white and brown pre-adipocytes were isolated from mouse inguinal WAT and interscapular BAT as described before.^13^ Briefly, after collagenase (0.2%) digestion, filtration and centrifugation (1500 r.p.m., 5 min), the stromal vascular fraction/pre-adipose cells were re-suspended in basal growth medium (Dulbecco’s modified Eagle’s medium, DMEM, supplemented with 10% fetal bovine serum (FBS), 1% penicillin and streptomycin), and cultured at 37 °C in a humidified atmosphere containing 5% CO_2_ and 95% air. For adipocyte differentiation, fully confluent pre-adipose cells (defined as day 0) were treated for 2 days with induction medium (growth medium supplemented with 0.5 μg ml^−1^ insulin, 0.5 mm isobutylmethylxanthine (IBMX), 100 nm dexamethasone, 1 μm rosiglitazone). After 48 h, the cells were incubated for another 2 days with growth medium containing insulin, and thereafter, the medium was replenished every other day for the next 4–6 days before conducting the experiments (days 8–10).

The human pre-adipose cells (Zen-Bio, Inc., Research Triangle Park, NC, USA) collected from subcutaneous WAT of non-diabetic subject (body mas index 25–29.9) were grown till confluence in DMEM/ F-12 containing 10% FBS. Adipocyte differentiation was then induced similarly as the previously reported.^18,23^ Briefly, the cells (defined as day 0) were treated for 4 days with serum-free differentiation medium (DMEM/F-12 containing 33 μm biotin, 17 μm pantothenate, 10 μg ml^−1^ transferrin, 1 nm triiodothyronine (T3), 0.5 μg ml^−1^ insulin, 0.5 mm IBMX, 100 nm dexamethasone and 1 μm rosiglitazone). The cells were then cultured with the serum-free growth medium (DMEM/F-12 containing insulin and dexamethasone) for 10–12 days, with medium changes every 3 days, before conducting the experiments (days 14–16).

Adipocyte differentiation was confirmed by Oil Red O staining, visual appearance of lipid droplets, as well as the immunoblot analyses of adipocyte-specific protein (for example, adipocyte protein 2 (aP2)). To quantify the number of white adipocytes with multilocular appearance (adipocytes with the largest lipid droplet (LD) smaller than 10–15 μm in diameter) or brown adipocytes (classical brown adipocytes differentiated from brown pre-adipose cells of interscapular BAT), 2–3 bright-field images (100–400×) were randomly acquired from each treatment using an Olympus microscope (IX71) equipped with a digital camera (OlympusE330). Cells were then counted manually from the bright-field images using the ImageJ cell counter plugin. All experiments were independently repeated at least 3–4 times using the primary cells from different donors. Unless otherwise stated, white adipocytes differentiated from primary mouse pre-adipose cells of inguinal WAT were used in this study. AngII, Ang(1–7), ZD7155, PD123319, CGP42112, CL316,243, UO126, 10-DEBC and Dorsomorphin were purchased from Tocris Bioscience; Captopril, TAPI-2 and A779 from Santa Cruz Biotechnology (Reston, VA, USA); DX600 from BioVision (Milpitas, CA, USA); and M024/C21 (C21) from Axon Medichem. All culture media, supplements and sera were from Life Technologies, Inc. (ThermoFisher Scientific, Waltham, MA, USA). All other chemicals and reagents were from Sigma-Aldrich (St Louis, MO, USA).

### Animal studies

All experimental protocols were approved by the SingHealth Research Facilities Institutional Animal Care and Use Committee (Singapore). Mice (C57BL/6J, 8–10 weeks old, male), housed in light- and temperature-controlled facility (12-h light/12-h dark cycle, 22 °C), were allowed free access to standard laboratory food and water. Mice were divided into ZD7155, M024/C21 or saline (as control) -treated groups. Based on the previous studies, mice were subjected to intraperitoneal injection of ZD7155 (1 mg kg^−1^ day) or M024/C21 (0.1 mg kg^−1 ^per day) dissolved in 100 μl of phosphate buffer saline (PBS) for 14 days.^20,24^ Body surface temperatures (which correlates to body core temperature^25^ and oxygen consumption rate)^26^ were monitored at room temperature (RT) by taking the thermographic images of mice using infrared thermal imaging camera (Flir T420; the thermal sensitivity, <0.045 °C and the spectral range, 7.5–13 μm) and analyzing with a FLIR-Tools software (FLIR System Inc, Wilsonville, OR, USA).

The mice sera were also collected for further analyses, before mice were killed by a lethal dose of carbondioxide. After measuring the weights of body and fat tissues (inguinal WAT, IgWAT; epididymal WAT, EpiWAT; interscapular BAT), IgWAT were removed and homogenized in radioimmuno-precipitation assay buffer (containing protease inhibitor mixture, Roche Applied Science, Indianapolis, IN, USA) for further immunoblot analyses. For morphological analysis of adipose tissues, both IgWAT and interscapular BAT were collected and fixed with 4% paraformaldehyde solution (overnight, at RT), washed with PBS, and immersed in 30% sucrose solution to cryoprotect the tissues (24 h, at RT). After embedding in FSC22 Frozen Section Media (Leica Microsystem, Buffalo Grove, IL, USA), cryosections (10–20 μm thick) were prepared using a cryostat (CM1950, Leica Microsystems), and images were taken using an Olympus microscope equipped with a digital camera.

### siRNA silencing

For gene silencing of ACE2 (*ACE2*), AT1R (*AGTR1*) or AT2R (*AGTR2*), adipose cells (day 4 after induction of differentiation) were transfected with a mouse ACE2 siRNA (sc-41401), AT1R siRNA (sc-29751), AT2R siRNA (sc-29753) or control siRNA (sc-37007) (Santa Cruz Biotechnology) as previously described.^13,18^ Specifically, adipose cells were incubated with Opti-MEM containing a complex of Lipofectamine-RNAiMAX transfection reagent (0.5% (v/v), Life Technologies, ThermoFisher Scientific) with siRNAs (25 nm) for 4 h, followed by the addition of grown medium and incubated for another 2–3 days. A mouse ERK1 siRNA (sc-29308), ERK2 siRNA (sc-35336), Akt1/2 siRNA (sc-43610) or AMPKα1/2 siRNA (sc-45313) were used for gene silencing of *MAPK3, MAPK1, AKT1/2* or *PRKAA1/2* in adipose cells. Knocking down of protein expressions were confirmed by immunoblot analyses.^13,18^

### Western blot analyses

The cells were washed with ice-cold PBS and collected in M-PER Mammalian Protein Extraction Reagent (containing Halt protease and phosphatase inhibitor cocktail, ThermoFisher Scientific). After a brief vortex and centrifugation at 4 °C, the supernatant was collected and the protein concentration was measured by a bicinchoninic acid (BCA) protein assay (ThermoFisher Scientific). Each sample with equal amount of proteins (as the loading control) was separated on 12% SDS-PAGE before transferred onto a nitrocellulose membrane. After blocking with Superblock blocking buffer at RT (2 h), membranes were incubated for 12 h with specific primary antibody (1: 100–400 dilutions) in Tris-buffered saline-Tween solution- TBST. Membranes were then washed three times with TBST (15 min each), and incubated for 6 h with horseradish peroxidase-conjugated secondary antibody (1: 2000–4000), followed by washing again with TBST for three times. The protein bands were detected in a G:BOX Chemi XT4 imaging system (Syngene, Frederick, MD, USA) using SuperSignal West Pico Chemiluminescent Substrate (ThermoFisher Scientific). At least 3–4 different experiments were performed separately using primary cells from different donors. Antibodies against UCP1 (sc-6529), CIDE-A (sc-366814), CITED1 (MSG1, sc-50743), aP2 (sc-18661), AT1R (sc-31181), AT2R (sc-9040), angiotensinogen (AGT, sc-7419), ACE (sc-20791), ACE2 (sc-20998), TACE (sc-6416), FAS (sc-20140), PPARγ (sc-7196), PRDM16 (sc-55697), thyroid hormone receptor TRβ1 (10822), Akt (sc-8312), phospho-Akt (Ser-474, sc-135651), AMPKα1/2 (sc-25792), phospho-AMPKα1/2 (Thr-172; sc-33524) and Actin (sc-1616) were purchased from Santa Cruz Biotech (Dallas, TX, USA). Antibodies against UCP1 (PA1–24894), PRDM16 (PA5-20872) and TRα1 (PA1-211A) were obtained from ThermoFisher Scientific. Antibodies against ERK1/2 (cat.no.085) and phospho-ERK1/2 (cat.no.3441) were from Biovision.

### Assessments on serological parameters

Serum adiponectin, TNFα and insulin levels in mice were analyzed by a mouse adiponectin ELISA kit (ThermoFisher Scientific), a mouse TNFα ELISA kit (Sigma-Aldrich) and a mouse insulin ELISA kit (ThermoFisher Scientific), respectively, according to manufacturer’s protocols. Serum levels of free fatty acids, triglycerides and glucose were measured by standard assay kits from Biovision. Serum cholesterol level was determined by a cholesterol quantitation kit (Sigma-Aldrich).

### Oxygen consumption rate assay

Oxygen consumption was analyzed using oxygen consumption rate assay kit from Cayman Chemical (MitoXpress-Xtra HS method) according to manufacturer’s instructions. Briefly, adipocytes grown in 96-well plates were treated with 10 μl MitoXpress-Xtra (a phosphorescent oxygen-sensitive probe whose signal increases overtime when oxygen in solution is depleting) immediately before measuring the fluorescence intensity (Ex/Em: 380 nm/650 nm, 0.5 min interval) using a time-resolved fluorescence plate reader (SpectraMax M5, Molecular Devices, Sunnyvale, CA, USA). Measurement was performed at 37 °C under a sealed environment by overlaying with 100 μl of HS Mineral Oil to limit the exchange of oxygen. Oxygen-consuming glucose oxidase and a potent mitochondrial uncoupling agent FCCP (4-trifluoromethoxy- carbonyl cyanide phenylhydrazone) was used as a reference.

### Confocal microscopy

Adipocytes grown on Lab-Tek (ThermoFisher Scientific) chambered cover-glass were fixed with 3.7% formaldehyde in PBS (15 min, RT) before washing twice with ice-cold PBS. In some experiments, the cells were permeabilized with 0.1% Triton X-100 (10 min, RT) and washed again with PBS. After being blocked with 1% bovine albumin (BSA) in PBST (PBS with 0.1% tween 20) (1 h, RT), the cells were incubated overnight (4 °C) in PBST containing 1% BSA and primary antibody (goat AT1R-IgG, rabbit AT2R-IgG, rabbit UCP1-IgG, goat TR β1-IgG, rabbit TRα1-IgG). After washing 3 times with PBS, the cells were then incubated in secondary antibody (donkey anti-goat IgG-CruzFluor594, sc-362275; bovine anti-rabbit IgG-CruzFluor488, sc-362260; donkey anti-goat IgG-PerCP-Cy5.5, sc-45102; goat anti-rabbit IgG-Atto647NHS, Sigma-Aldrich) (2 h, RT), followed by washing 3 times with PBS. The cells were imaged using a Confocal Laser Scanning Microscope (LSM 710, Carl Zeiss GMbH). Hoechst 33342 (NucBlue Live ReadyProbes Reagent, Life Technologies, ThermoFisher Scientific) was used to stain the nuclei. To reveal the mitochondrial membrane potential, adipocytes were incubated with the growth medium containing 0.2 μM MitoTracker Red FM (Life Technologies, ThermoFisher Scientific) (Ex/Em: 581 nm/644 nm) for 15 min (at 37 °C with 5% CO_2_ in a humidified incubator), before live-cell imaging with LSM710 (Carl Zeiss, Germany). MitoTracker Red is widely used to analyze membrane potential in living cells, since it is selectively localized in mitochondria while its accumulation is dependent on transmembrane potential.^13,27,28^ At least three independent experiments were performed using the primary cells from different donors.

### Statistical analyses

Data were expressed as mean±s.e.m. from at least 3–4 independently performed experiments. Statistical differences between two means were assessed using unpaired Student’s *t*-test. One-way analysis of variance (followed by Tukey's multiple comparisons test) was used to compare differences among multiple groups. A *P*-value <0.05 was considered statistically significant.

## Results

### AngII induces the browning characteristics in mouse white adipocytes via AT2R

**Figure 1:**
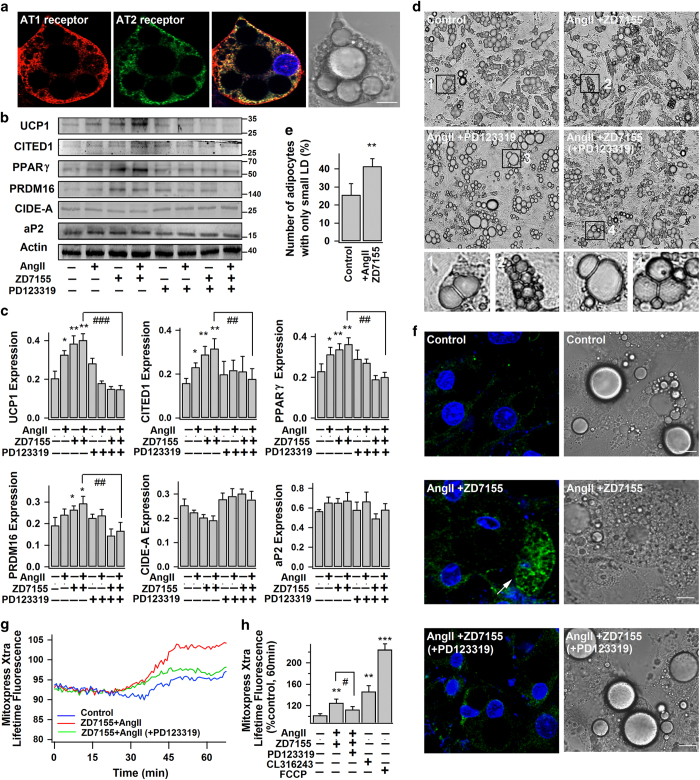
AngII–AT2R induces the browning characteristics in white adipocytes. (**a**) The representative confocal images of immunostained AT1R and AT2R in white adipocytes, their corresponding merged and bright-field images (from three different experiments). Scale bars=10 μm. (**b**–**h**) Mouse white adipocytes were treated without (−) (control) or with (+) 100 nm AngII, 1 μM ZD7155 or 1 μM PD123319 for 4 days. (**b** and **c**) The representative immunoblots of protein expressions in adipocytes: UCP1 (~33 kDa), CITED1 (~30 kDa), PPARγ (~57 kDa), PRDM16 (~150 kDa), CIDE-A (~26 kDa), aP2 (~16 kDa) and actin (~42 kDa); and the statistics (mean±s.e.m., *n*=4) of blot densities normalized to that of actin. (**d** and **e**) The representative bright-field images of differently treated adipocytes (from 3 different experiments). The percentage of adipocytes with only small LDs (the largest LD smaller than 10–15 μm in diameter) (mean±s.e.m.; *n*=10 images from three different experiments) is shown in **e**. (**f**) Confocal images of immunostained UCP1 in adipocytes, and their corresponding bright-field images. (Arrow shows the intense staining of UCP1 in brown-like adipocyte; from three different experiments) Scale bars=10 μm. (**g** and **h**) Lifetime fluorescence value of MitoXpress-Xtra assessed as an indication of oxygen consumption rate in adipocytes. The real time-responses of average fluorescence value and the statistics (at 60 min, *n*=6, from 2–3 different experiments) are shown. CL316,243 (1 μm, 4 days) and FCCP (1 μm, added immediately before the experiment) were used as positive controls. Data represent mean±s.e.m. *P<0.05, **P<0.01, ***P<0.001 versus control; ^#^*P*<0.05, ^##^*P*<0.01, ^###^*P*<0.001 between indicated pairs.

It has been known that AngII stimulates sympathetic neurotransmission to adipose tissue, while catecholamine released from sympathetic nerves are considered major stimulating factors for browning of white adipose tissues.^29^ As both AngII and AngII receptors (AT1R and AT2R) are expressed in white adipocytes (Figure 1a),^15,16^ we sought to investigate whether AngII has a direct role in browning of white adipocytes.

As shown in Figures 1b and c, mouse white adipocytes have negligible amount of uncoupling protein 1 (UCP1, brown adipocyte-specific protein and thermogenic marker), which is consistent with our previous finding and other studies.^13,30^ Nevertheless, UCP1 level was significantly increased in AngII-treated white adipocytes. Expression of beige adipocyte-specific marker, CITED1,^7^ was also upregulated with AngII treatment. Blockage of AT1R (by ZD7155, a selective non-peptide AT1R antagonist) largely enhanced AngII-induced UCP1 and CITED1 expressions, and such enhancement was effectively prevented by co-application of PD123319 (a selective non-peptide AT2R antagonist). These observations suggest the stimulatory role of AT2R and inhibitory role of AT1R on white adipose browning in response to paracrine or endocrine AngII stimulation. Furthermore, blockage of AT1R alone, but not AT2R, increased UCP1 and CITED1 expressions in white adipocytes (Figures 1b and c, Supplementary Figure S1). It implies that under basal condition the autocrine browning stimulation of AT2R by the endogenously produced AngII is suppressed by autocrine activation of AT1R.

Consistently, expressions of PPARγ and PRDM16 (major transcription factors known to promote expressions of genes required for adipose browning and thermogenesis)^31^ were significantly increased by AngII–AT2R interaction, and such upregulations could be rectified by co-treatment with AT2R antagonist (Figures 1b and c). Also, brown adipocyte-like features, namely multilocular appearance (multiple small lipid droplets, LDs) and dark-brown color due to high mitochondrial contents,^30^ were frequently observed in AngII+ZD7155 treated cells (allowing selective stimulation of AT2R), while the unilocular appearance (large central LD) was mostly found in the control and AngII+PD123319 treated cells (Figure 1d). Figure 1e shows the increased proportion of multilocular adipose cells (adipocytes with the largest LD smaller than 10–15 μm) after AngII+ZD7155 treatment. We further found that the AngII+ZD7155 treated adipocytes with multilocular appearance were usually UCP1-expressing cells (as evidenced by the immunostaining of UCP1), whereas the multilocular ones found in control and AngII+PD123319 treated groups were not UCP1 expressing (Figure 1f, Supplementary Figure S2). In addition, AngII+ZD7155 treatment (AT2R stimulation) was associated with increased oxygen consumption rate (OCR) which reflects the mitochondrial respiratory activity and metabolic rate in adipocytes (Figures 1g and h). Simultaneous blockage of both AT1R and AT2R (AngII+ ZD7155+PD123319) effectively blocked OCR increase and induction of brown-like phenotype. All these suggest that AngII promotes the browning of white adipocytes by stimulating the expressions of PPARγ and PRDM16 via AT2R.

**Figure 2:**
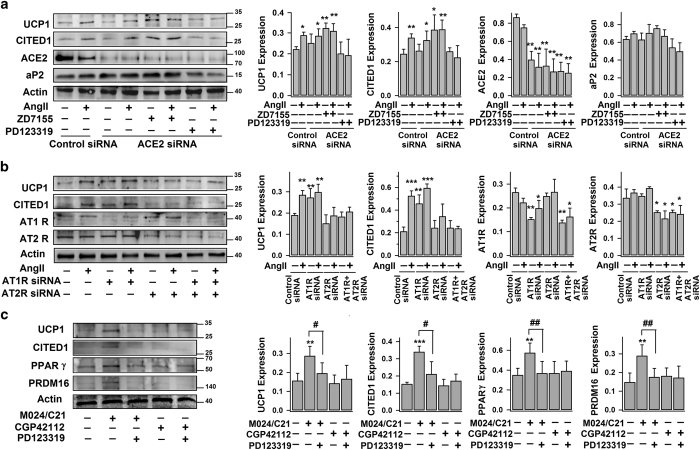
AT2R activation increases UCP1 and CITED1 expressions in white adipocytes. (**a** and **b**) Mouse white adipose cells (day 4) were transfected with control siRNAs, ACE2-siRNA, AT1R-siRNA or AT2R-siRNAs for 2–3 days, before treated without (−) (control) or with (+) AngII, ZD7155 or PD123319 for another 4 days. (**c**) Mouse white adipocytes were treated without (−) (control) or with (+) 100 nM M024/C21, 100 nM CGP42112 or PD123319 for 4 days. The representative immunoblots of protein expressions (UCP1, CITED1, ACE2, AT1R, AT2R, PPARγ, PRDM16, aP2 and actin), and the statistics (mean±s.e.m., *n*=4; normalized to actin densities) are shown accordingly. *P<0.05, **P<0.01, ***P<0.001 versus control; ^#^*P*<0.05 and ^##^*P*<0.01 between indicated pairs.

In adipocytes, AngII can be cleaved into Ang(1–7) by the membrane-bound ectoenzyme—angiotensin-converting enzyme-2 (ACE2).^16^ Although the main receptor of Ang(1–7) is Mas receptor, it can also activate AT2R.^32,33^ As shown in Supplementary Figure S3, exogenously application of Ang(1–7) is able to promote UCP1 expression in white adipocytes. But in the ACE2-knockdown adipocytes (using specific ACE2-siRNA), both AngII and AngII+ZD7155 treatments were still able to promote UCP1 and CITED1 expressions (Figure 2a). Similarly, AngII+ZD7155 treatment increased UCP1 and CITED1 expressions in adipocytes co-treated with DX600 (a potent ACE2 inhibitor) (Supplementary Figure S3). Therefore, despite the stimulatory effect of Ang(1–7), AngII-induced browning is through its direct interaction with AT2R instead of through ACE2-Ang(1–7) pathway.

Consistently with the experiments shown in Figures 1b and c using specific antagonists, activation of AT2R by AngII in AT1R knockdown cells increased both UCP1 and CITED1 expressions whereas AT1R activation in AT2R knockdown cells failed to do so (Figure 2b, Supplementary Figure S2). In addition, the expressions of UCP1 and CITED1 in the AT1R knockdown cells were elevated, confirming our view that AT1R suppresses the endogenous AngII–AT2R browning pathway. This is consistent with a recent study showing the upregulation of thermogenic gene expression in WAT of AT1R knockout mice.^34^ Finally, we observed that M024/C21 (also known as C21; a selective non-peptide AT2R agonist),^35–37^ but not CGP42112 (a selective AT2R ligand), stimulated the expressions of UCP1, CITED1, PPARγ and PRDM16; and these M024/C21-induced upregulations could be effectively abolished by co-incubation with AT2R antagonist—PD123319 (Figure 2c). Taken together of these multiple lines of evidence, we conclude that AngII stimulates browning by directly activating AT2R but at basal state this endogenous autocrine stimulation is suppressed by AT1R.

### AngII–AT2R-induced white adipocyte browning is dependent on ERK1/2, Akt and AMPK signaling pathways

We and others previously revealed that AngII-AT1R interaction triggered MEK/ERK pathway in white adipocytes.^38,39^ In contrast, a number of studies in different cell types (for example, neurons) showed that AT2R activation decreases ERK1/2 activity via activation of phosphatases, such as MAP kinase phosphatase-1 (MKP-1).^35^ Studies have also showed that AngII regulates Akt and AMPK signaling pathways in adipocytes and other cell types.^40,41^

**Figure 3:**
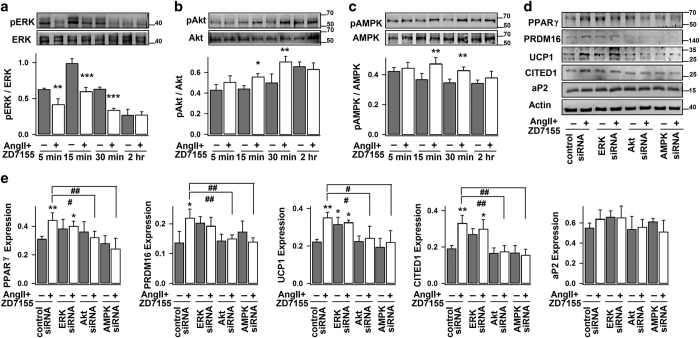
Signaling pathways underlying the AngII–AT2R-induced browning of white adipocytes. (**a**–**c**) Time course of the phosphorylation and total protein levels of ERK1/2, Akt and AMPK in mouse white adipocytes, without (−) (control) or with (+) exposure to AngII and ZD7155. Top panels, representative immunoblots; bottom panels, statistics (mean±s.e.m., *n*=3) of the optical density ratio between pERK and ERK, pAkt and Akt, or pAMPK and AMPK. (**d** and **e**) Mouse white adipose cells (day 4) were transfected with control siRNA, ERK1/2-siRNAs, Akt-siRNA or AMPK-siRNAs for 2–3 days before treated without (−) (control) or with (+) AngII and ZD7155 for 4 days. The representative immunoblots and the statistics (mean±s.e.m., *n*=4; normalized to actin densities) are shown accordingly. *P<0.05, **P<0.01, ***P<0.001 versus control; ^#^*P*<0.05 and ^##^*P*<0.01 between indicated pairs.

Here, we found that selective activation of AT2R by co-treatment with AngII and AT1R antagonist reduced ERK1/2 phosphorylation (in 5 min) but increased Akt and AMPK phosphorylation (in 15 and 30 min, respectively) in white adipocytes (Figures 3a–c). Similarly, M024/C21 treatment reduced ERK1/2 phosphorylation and increased Akt phosphorylation in white adipocytes, and such changes were effectively prevented by co-application of AT2R antagonist PD123319 (Supplementary Figure S4). It has also been demonstrated in adipocytes and other cell types that inhibition of ERK signaling (by either chemical inhibitor or knockdown of ERK) is accompanied by improved AMPK and Akt signaling.^40,42,43^ Hence, one may conclude that AngII–AT2R interaction rapidly thwarts ERK signaling which subsequently promotes the downstream Akt (and AMPK) pathway in white adipocytes.

Consistently, AngII–AT2R-induced expressions of PPARγ, PRDM16, UCP1 and CITED1 were concealed in both Akt and AMPK knockdown cells (but not in ERK1/2 knockdown cells) (Figures 3d and e). AngII–AT2R-induced expressions of UCP1 and CITED1 were also significantly abolished by the pharmacologic inhibition of Akt and AMPK signaling, but not of MEK/ERK signaling (Supplementary Figure S4). Recent studies have showed that both Akt and AMPK signaling are important for inducing brown-like phenotypes in WAT,^13,44^ whereas ERK signaling suppresses expression of genes controlling thermogenesis and browning of WAT in mice.^11,45^ Taken together, it may be concluded that AngII–AT2R-induced white adipose browning is, at least in part, dependent on ERK, Akt and AMPK signaling pathways.

### AngII–AT2R enhances brown adipogenesis

**Figure 4:**
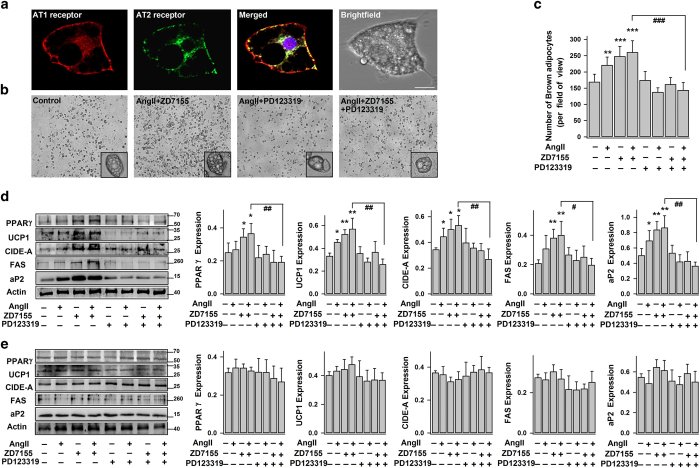
AngII–AT2R enhances brown adipocyte differentiation. (**a**) The representative confocal images of immunostained AT1R and AT2R in brown adipocytes, their corresponding merged and bright-field images (from 3 different experiments). Scale bars=10 μm. (**b**–**d**) Mouse brown pre-adipocytes (from interscapular BAT) (day 0) were induced to differentiate into brown adipocytes without (−) (control) or (+) with exposure to AngII, ZD7155 or PD123319. The representative bright-field images and number of brown adipocytes (multiloculated cells, day 8) per field-of-view (100x) (mean±s.e.m.; *n*=8 images from 3–4 separate experiments) are shown accordingly. (**d**) The representative immunoblots of protein expressions (PPARγ, UCP1, CIDE-A, FAS, aP2, actin) (day 8) and the statistics (mean±s.e.m., *n*=4) of the blot densities normalized to actin density. (**e**) Brown adipocytes (day 8) were treated for 4 days, without (−) (control) or (+) with AngII, ZD7155 or PD123319. The representative immunoblots and the statistics (mean±s.e.m., *n*=4; normalized to actin densities) are shown accordingly. *P<0.05, **P<0.01 versus control; ^#^*P*<0.05, ^##^*P*<0.01 and ^###^*P*<0.001 between indicated pairs.

Angiotensinogen (the sole precursor of AngII) is expressed in interscapular brown adipose tissue (classical BAT).^46^ Here, we found that both AT1R and AT2R are expressed in brown adipocytes isolated from interscapular BAT (Figure 4a). We conceive that, in addition to stimulation of white adipocyte browning, AngII–AT2R signaling may also play regulatory roles in brown adipogenesis (brown pre-adipocytes of interscapular BAT differentiate into mature brown adipocytes).

As shown in Figures 4b and c, exogenous application of AngII, ZD7155 or AngII+ZD7155 (since the induction of brown pre-adipocyte differentiation at day 0) significantly increased the number of differentiated brown adipocytes (as indicated by their multilocular appearance). Consistently, these treatments largely stimulated the expressions of adipogenic transcription factor (PPARγ), brown adipocyte-specific proteins (UCP1 and CIDE-A) and late markers of differentiated adipocytes (aP2 and FAS; Figure 4d). Blockage of AT2R (using PD123319) mostly removed the stimulatory effects of AngII and/or ZD7155 on brown adipogensis. On the other hand, AngII, ZD7155 and PD123319 (treatment at day 8) exerted no significant effect on the expressions of PPARγ, UCP1 (a key thermogenic protein), CIDE-A, aP2 and FAS in mature brown adipocytes (Figure 4e). Therefore, AT2R activation (induced by either exogenously applied AngII or endogenously produced AngII) has stimulatory role in brown adipogenesis, but doesn’t significantly affect the basal activities of mature brown adipocytes.

### AT2R activation promotes WAT browning and reduces WAT mass *in vivo*

**Figure 5:**
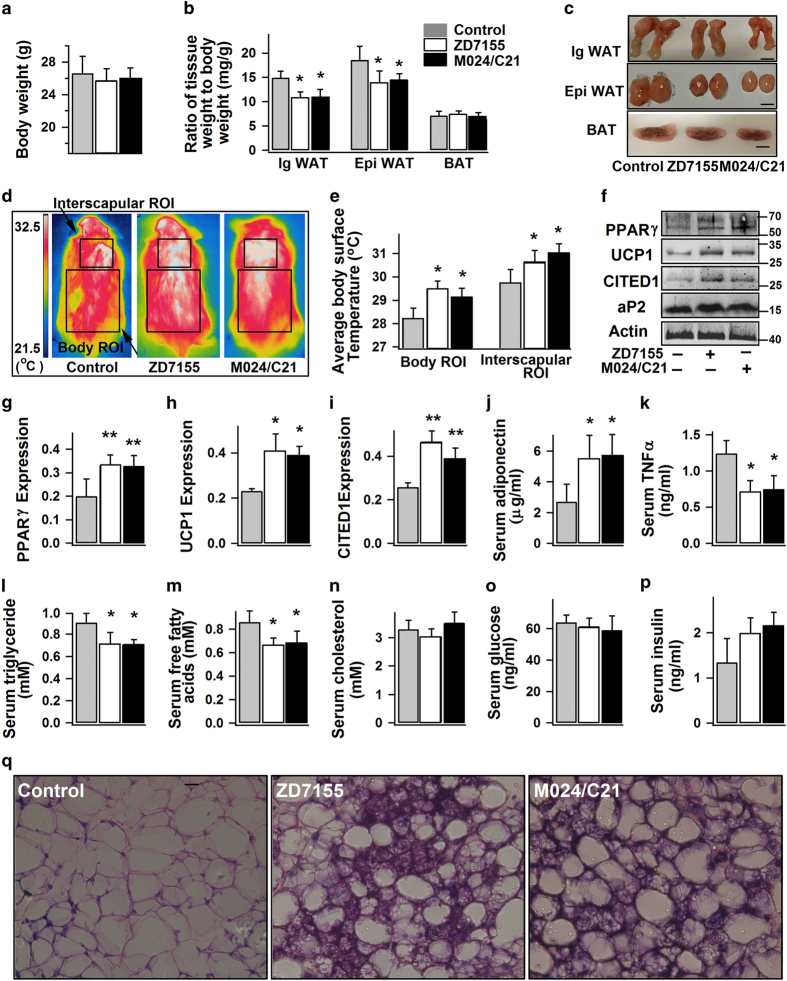
AT2R activation stimulates WAT browning and increases body surface temperature in mice. 8–10 week old mice were given intraperitoneal injection of normal saline (100 μl, control), ZD7155 (1 mg kg^−1^ per day) or M024/C21 (0.1 mg kg^−1^ per day) for 14 days. (**a**–**c**) Average body weights, and weights of inguinal WAT (IgWAT), epididymal WAT (EpiWAT) and interscapular BAT relative to the total body weights (*n*=6 mice per group). The representative images of IgWAT, EpiWAT and BAT isolated from the differently treated mice are shown in (**c**). Scale bars=4 mm. (**d** and **e**) Infrared thermographic analysis of body surface temperature in differently treated mice. The representative images and the statistical analyses of the average body surface temperature within a region of interest (ROI), at body or interscapular area, are shown accordingly (*n*=6 mice per group). (**f**–**i**) The representative immunoblots of protein expression in IgWAT from differently treated mice, and the statistics (*n*=4 mice per group; normalized to actin densities) are shown accordingly. (**j**–**p**) Quantification of serum levels of adiponectin, TNFα, triglycerides, free fatty acids, cholesterol, glucose and insulin from differently treated mice (*n*=4 mice per group). (**q**) The representative hematoxylin and eosin staining images of IgWAT from the differently treated mice (400×; from four mice per group). Data are presented as mean±s.e.m., *P<0.05, **P<0.01 versus control.

To further investigate the role of AT2R in WAT browning *in vivo*, we gave mice intraperitoneal injection of ZD7155 (specific AT1R antagonist that permits selective AT2R activation by endogenously produced AngII) or M024/C21 (specific AT2R agonist that directly activates AT2R). No significant difference in body weight (Figure 5a) or food intake (data not shown) was observed between the control group (saline injection) and ZD7155 or M024/C21 treated group. Interestingly, WAT mass (both inguinal and epididymal WAT) was obviously reduced in both ZD7155 and M024/C21 treated mice, whereas the weight of interscapular BAT was similar to that of the control (Figures 5b and c). In addition, similar to the mice treated with CL316, 243 (a specific β3-adrenergic receptor agonist; as a positive control; Supplementary Figure S5), the body surface temperature was elevated in both ZD7155 and M024/C21 treated mice (Figures 5d and e), indicating increased body metabolic rate and heat production. Consistently, both ZD7155 and M024/C21 treatments similarly promoted the browning of WAT, as evidenced by the increased expressions of PPARγ, UCP1 and CITED1 (Figures 5f–i) in inguinal WAT isolated from the treated mice. Furthermore, the histological appearance of multilocular brown-like adipose cells in inguinal WAT of ZD7155 or M024/C21 treated mice unambiguously confirms their browning effects on WAT (Figure 5q, Supplementary Figure S6). M024/C21-induced PPARγ expression in adipose tissue and decrease of WAT weight are consistent with the previous studies.^20,47^ Increased surface temperature around the interscapular area (which reflects BAT-derived thermogenesis)^26^ in ZD7155 or M024/C21 treated mice (Figures 5d and e) is also consistent with our results of increased brown adipogenesis induced by AT2R activation (Figure 4).

Furthermore, mice receiving either ZD7155 or M024/C21 showed a significant increase of serum adiponectin concentration, and reduction of serum levels of TNFα, triglycerides, and free fatty acid (Figures 5j–m). On the other hand, there was no significant difference in the serum levels of cholesterol (Figure 5n), glucose (Figure 5o), and insulin (Figure 5p) between the groups. In sum, the *in vivo* experiments further confirm the stimulatory roles of AT2R activation on WAT browning, which is accompanied with whole body energy metabolism (as indicated by the reduction of WAT mass and serum levels of triglycerides and free fatty acids) and thermogenesis (as indicated by the raised body temperature).

### AngII–AT2R increases UCP1 expression and basal metabolic activity in human white adipocytes

**Figure 6:**
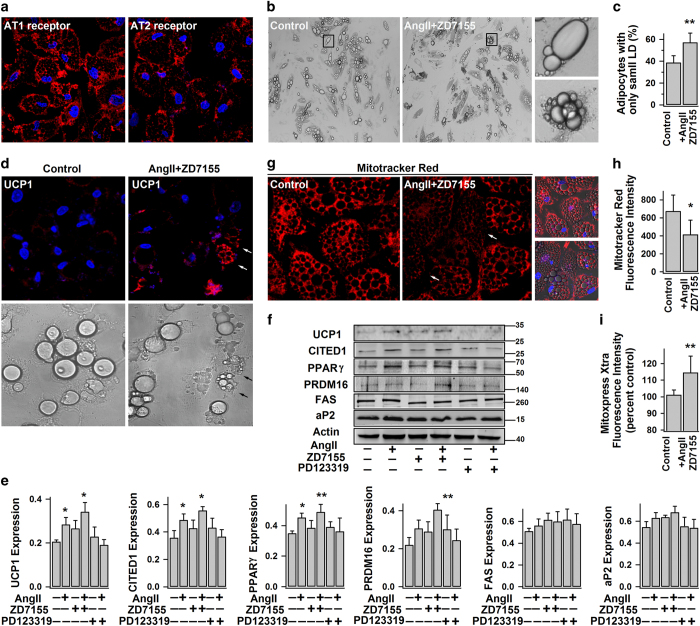
AngII–AT2R increases UCP1 expression and basal metabolic activity in human white adipocytes. (**a**) The representative confocal images of immunostained AT1R and AT2R in human white adipocytes (day 14) (from three different experiments). (**b**–**e**) Human white adipocytes were treated without (−) (control) or with (+) AngII, ZD7155 or PD123319 for 4–5 days. (**b** and **c**) The representative bright-field images of differently treated adipocytes (from 3 different experiments). The percentage of adipocytes with only small LDs (the largest LD smaller than 10–15 μm in diameter) (mean±s.e.m.; *n*=10 images from 3 different experiments), is shown in (**c**). (**d**) The representative confocal images of immunostained UCP1 in adipocytes and their correspondingly bright-field images (from 3 different experiments; arrow shows the intense UCP1staining in beige adipocyte). (**e** and **f**) Shown are the representative immunoblots of protein expressions and the statistics (mean±s.e.m., *n*=4, normalized to actin density). (**g** and **h**) The representative confocal images of adipocytes showing the changes of mitochondrial membrane potential (detected by MitoTracker Red), and the statistics of fluorescence intensity (mean±s.e.m., *n*=12, from 3 different experiments). (**i**) Lifetime fluorescence value of MitoXpress-Xtra assessed as an indication of oxygen consumption rate (at 60 min; mean±s.e.m., *n*=6, from 2–3 separate experiments) in adipocytes. *P<0.05 and **P<0.01 versus control.

To test the human relevance of our discovery in mouse white adipocytes, we also conducted the experiments using human white adipocytes. We first confirmed the presence of both AT1R and AT2R in human white adipocytes (Figure 6a), in agreement with our previous work and other studies.^18,48,49^ Similarly, AT2R activation (AngII+ZD7155) increased number of multilocular adipose cells (adipocytes with the largest LD smaller than 10–15 μm; Figures 6b and c) and brown-like phenotypes in human white adipocytes, namely the common appearance of UCP1-positive dark-brown multilocular beige adipocytes (Figures 6b and d). As expected, the expressions of UCP1, CITED1, PPARγ and PRDM16 proteins were also boosted in the AT2R-activated white adipocytes (AngII+ ZD7155), but not in AT1R-activated cells (AngII+PD123319; Figures 6e and f).

UCP1 promotes mitochondrial respiration and heat generation by decreasing the proton gradient generated in oxidative phosphorylation, hence the mitochondrial membrane potential.^50^ In line with the increase of UCP1 expression, AT2R activation lowered the mitochondrial membrane potential as evidenced by the decreased fluorescence intensity of MitoTracker Red staining (Figures 6g and h) and increased the basal activity of mitochondrial respiration as indicated by the increased oxygen consumption rate (Figure 6i). Taken together with the results shown in Figures 1,2,3,4,5, one may conclude that AngII–AT2R signaling promotes white adipose browning in both human and rodents.

### AT2R is upregulated by thyroid hormone T3 and plays a significant role in T3-induced white adipose browning

All components of angiotensin system (including ACE, ACE2, AT1R and AT2R) are present in white adipocytes and closely linked to the development of obesity and related metabolic disorders.^16,17^ As white adipocytes are inducible into brown-like adipocytes in response to various activators, especially cold exposure and thyroid hormone, we have investigated whether adipose angiotensin system (RAS) acts the mediator.

**Figure 7:**
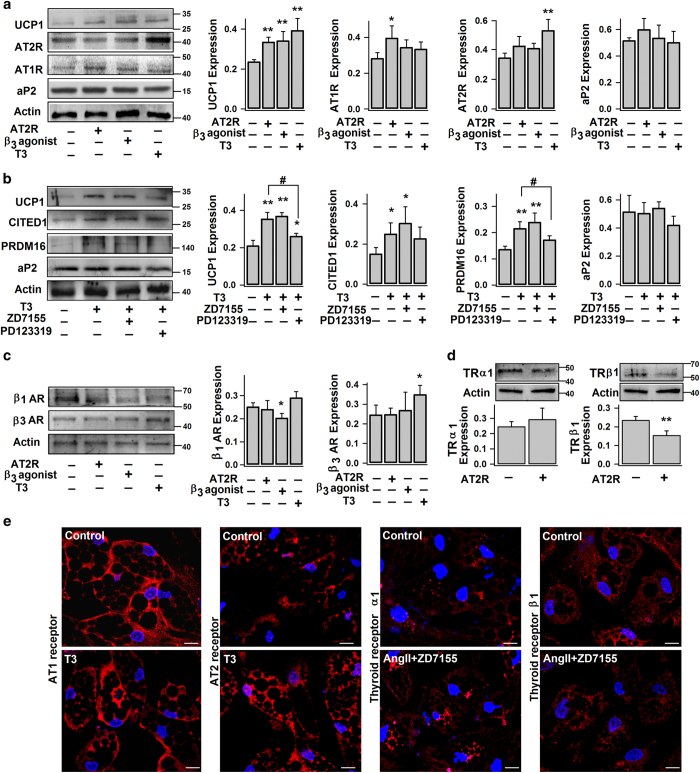
AT2R expression in white adipocyte is upregulated by thyroid hormone T3. Human white adipocytes (day 14) were treated without (−) (control) or with (+) AngII, ZD7155, PD123319, 1 μM CL316,243 or 50 nM thyroid hormone T3 for 4–5 days. (**a**–**d**) The representative immunoblots of protein expressions (UCP1, CITED1, PRDM16, AT1R (~41 kDa), AT2R (~43 kDa), β1-adrenergic receptor (β1AR, ~65 kDa), β3AR (~44 kDa), thyroid receptor α1 (TRα1, ~48 kDa), TRβ1 (~53 kDa), aP2 and actin) and the statistics (mean±s.e.m., *n*=4; normalized to actin densities) are shown accordingly. (**e**) The representative confocal images of immunostained AT1R, AT2R, TRα and TRβ1 in differently treated white adipocytes (from three separate experiments). Scale bars=10 μm. *P<0.05, **P<0.01 versus control;^ #^*P*<0.05 between indicated pairs.

As shown in Figure 7a, selective activation of either AT2R (by AngII while blocking AT1R), β3-adrenergic receptor (by CL316,243) or thyroid nuclear receptors (by thyroid hormone T3) significantly upregulated UCP1 expression in human white adipocytes. Interestingly, T3 treatment stimulated the expression of AT2R (Figures 7a and e) but not the other RAS components (AT1R, ACE, ACE2, TACE and AGT; Figure 7a and Supplementary Figure S7), implying that AT2R has a critical role in T3’s browning effects. This notion is corroborated by the observations that T3-induced increase of browning markers (UCP1, CITED1 and PRDM16) was partially reversed by blocking AT2R (Figure 7b). In contrast, β3-adrenergic receptor agonist had no significant effects on the expression of angiotensin system including AT2R (Figure 7a and Supplementary Figure S7).

Activation of AT2R, on the other hand, reduced the expression of thyroid hormone receptor β1 (TRβ1, the specific TR isoform responsible for the upregulation of UCP1 in brown adipocytes), but without significant effect on the expression TRα1 (major TR isoform present in adipocytes) and β adrenergic receptors (Figures 7c and d). The observation of AT2R-suppressed TRβ1 expression may explain why there was no synergistic effect on adipose browning when AT2R and TR were simultaneously activated (Figure 7b). Herein, we reveal the intricate crosstalks between AT1R, AT2R and TR in white adipocyte browning.

## Discussion

**Figure 8:**
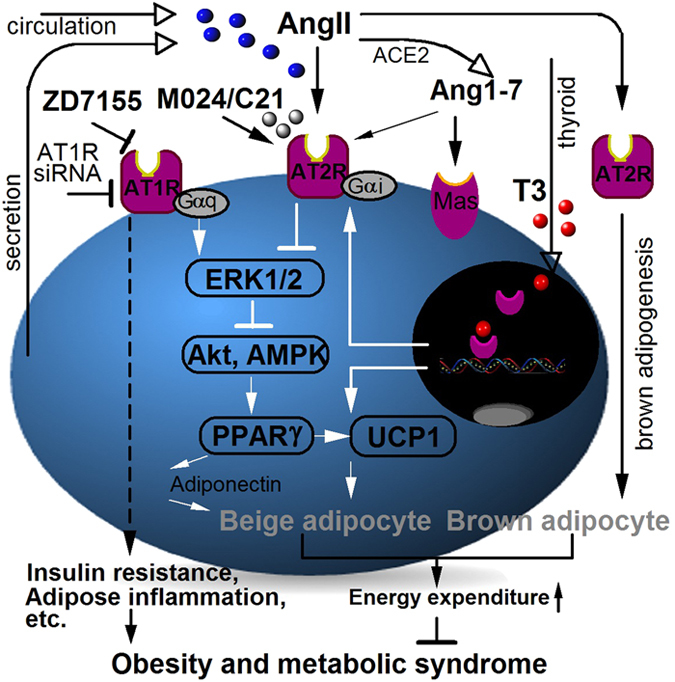
Illustration of AT2R-induced adipose tissue browning.

With the recent identification of WAT remodeling into brown-like adipose tissue (that is, beige or brite adipocytes), there is enormous interest in discovering how to enhance white adipose browning as a therapeutic strategy for treating obesity. WAT Browning not only suppresses obesity through increased energy expenditure, but also reduces adverse effects of excess WAT such as insulin resistance, cardiovascular complications, etc.^6,8^ Recent evidences suggest that adipokines secreted by adipose tissue act as the autocrine and paracrine signals to regulate its own browning.^11–13^ In this study, we provide *in vitro* and *in vivo* evidence that AngII, which is an adipokine secreted by adipocytes or endothelial cells in adipose tissue and an endocrine hormone supplying to adipose via circulation, promotes white adipose browning and brown adipogensis via activation of AT2R. Figure 8 summaries our major findings, some of which are also supported by ours and others’ previous studies.

Adipocytes, a major contributor to plasma AngII level, express both AngII receptors (AT1R and AT2R) with the majority of AT1R subtype.^15^ Normally AT1R functions to suppress adipogenesis, lipolysis and glucose uptake in adipocytes. But in obesity, elevated AngII level together with overexpression of adipose AT1R is one of the main mechanisms that links obesity and metabolic disorders (for example, insulin resistance).^17,22^ For example, AT1R-exerted suppression of adipogenesis ^18,38^ causes excessive lipid accumulation; thus converting adipocytes into hypertrophic and insulin-resistant state in obese rodents.^19,51^ In obesity, AngII-AT1R increases adipose production of several pro-inflammatory cytokines, particularly TNFα,^17,52^ which in turn suppress white adipocyte browning^11^ and trigger apoptosis of brown adipocytes.^53^ Reduced BAT mass (hence energy expenditure) further exacerbates the growth of WAT for excess energy storage.^5,6^ On the other hand, AT2R activation is able to enhance both white^18,21^ and brown adipogenesis (Figure 4). AT2R stimulates white adipocyte browning through ERK1/2, Akt and AMPK signaling pathways (Figure 3), leading to increased expressions of transcriptional factors PPARγ and PRDM16, and subsequently enhanced UCP1 expression and exhibition of brown-like phenotypes (Figures 1, 2, 5 and 6). AT2R also promotes adipose production of anti-inflammatory adipokines,^54^ including adiponectin (Figure 5j) which is known to be beneficial to WAT browning.^12^ As a result of increased number of brown and brown-like adipocytes, body energy expenditure increases (Figure 5d), and WAT depots are reduced (Figures 5b and c). Taken together, AT2R offers multiple beneficial effects to combat obesity. This notion is consistent with some previous studies. Specifically, it has been shown in mice that chronic AT2R activation using C21 causes an increase of white adipocyte differentiation and PPARγ expression, and a decrease in adiposity, adipose tissue inflammation and insulin resistance,^20,21,47^ while knockout of AT2R does the opposite.^55,56^ And resting metabolic rate, detected by direct calorimetry method, is significantly decreased in AT2R knockout mice.^57^

Browning of WAT can be induced by prolonged cold exposure and other stress conditions through the activation of sympathetic nervous system.^12,58^ Sympathetic norepinephrine activation of β-adrenergic receptor increases cAMP accumulation, which in turn induces UCP1 expression and brown-like phenotypes (for example, mitochondrial biogenesis) in white adipocytes.^6^ cAMP also stimulates expression and release of angiotensinogen (AGT), a precursor peptide of AngII, in human adipose cells.^59^ In fact, plasma AngII level increases markedly after cold exposure or under stress conditions (for example, exercise) in human.^60^ Plasma AngII level can also be increased by thyroid hormone T3, another well-known activator of adipose browning.^61^ Here, we found that T3 also stimulates adipose AT2R expression. It has been shown that hormone estrogen induced UCP1 expression is associated with increased AT2R expression in WAT.^47^ Evidently, AT2R is critically involved in the browning process induced by stresses or endocrine hormones.

Previous studies have revealed the autocrine and paracrine regulations by adipokines on self-modeling of adipose tissue including browning.^9,13,62^ TNFα, an adipokine known to inhibit white adipocyte browning, suppresses adipose expression of the precursor peptide of AngII (AGT).^63^ Apelin, an adipokine known to promote white adipose browning, inhibits AT1R by triggering heterodimerization between APJ receptor and AT1R.^64^ It has also been demonstrated that AT1R blockage (and AT2R activation) increases apelin production and decreases TNFα production in white adipocytes.^52,65^ All these observations, together with our findings here, suggest the central role of AT2R in autocrine and paracrine regulation of browning. Interestingly, AT2R activation may serve as the protective response to diet-induced obesity because high-fat diet stimulates adipose expression of AT2R.^47^

AngII–AT2R signaling can be exploited for obesity treatment. It has been reported that systemic infusion of AngII or AngII analog increases energy expenditure and reduces WAT mass in rodents.^66,67^ However, AngII infusion is usually associated with many systemic adverse effects produced by AT1R (for example, hypertension).^66^ Therefore, direct activation of AT2R by introducing exogenous selective agonists and making endogenous AngII-induced basal AT2R activity more prominent by inhibiting AT1R should be better strategies. Here, we show that both specific AT1R antagonist and AT2R agonist lead to brown-like phenotypes in white adipocytes, and reduction of fat mass as well as serum levels of free fatty acids and triglycerides. In summary, this study establishes the central roles of AT2R in white adipose browning and brown adipogenesis and suggests new strategies to combat obesity.
