# Author Correction: Angiotensin type 2 receptor activation promotes browning of white adipose tissue and brown adipogenesis

**DOI:** 10.1038/s41392-018-0014-9

**Published:** 2018-04-19

**Authors:** Aung Than, Shaohai Xu, Ru Li, Melvin Khee-Shing Leow, Lei Sun, Peng Chen

**Affiliations:** 1 School of Chemical and Biomedical Engineering, Nanyang Technological University, Singapore, Singapore; 2 Duke-NUS Graduate Medical School, Singapore, Singapore; 3 Endocrine and Diabetes, Tan Tock Seng Hospital, Singapore, Singapore

**Correction to:** *Signal Transduction and Targeted Therapy* 10.1038/sigtrans.2017.22; published online 23 June 2017

The original version of this article contained an error in author names, which was incorrectly given as ‘MelvinKhee-Shing Leow’, the correct name of the author is Melvin Khee-Shing Leow and the initials should be Leow MK.

This error has now been corrected in the HTML and PDF versions of the article.

