# Supplementary material for: Angiotensin type 2 receptor activation promotes browning of white adipose tissue and brown adipogenesis
Source: Signal Transduct Target Ther. 2017 Jun 23;2:17022–. doi: 10.1038/sigtrans.2017.22 (PMC5661636; doi:10.1038/sigtrans.2017.22)
Supplement: Supplementary Figures [file sigtrans201722-s1.doc]

**Supplementary information**

**
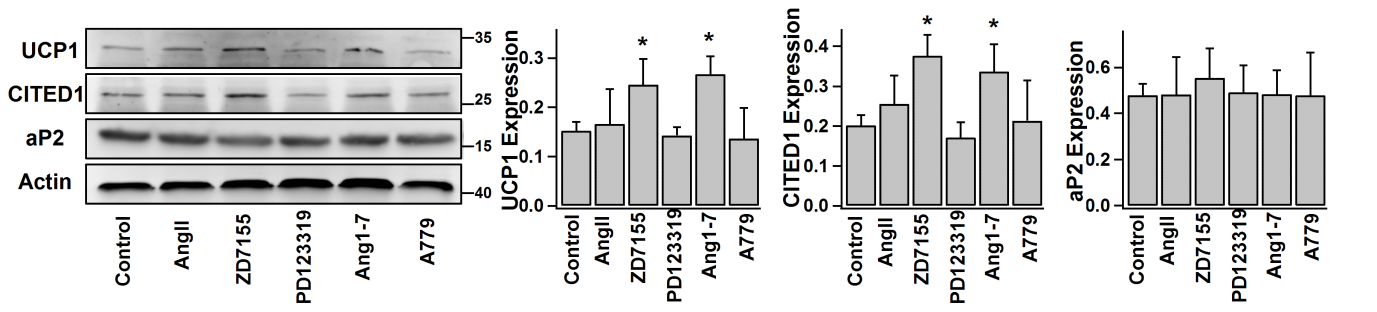
**

**Supplemental Figure 1S:** 3T3-L1 adipocytes (day8) were treated for 4 days, without (-) (control) or with (+) AngII, ZD7155, PD123319, 100 nM Ang(1-7) or 1 µM A779 (a selective Ang(1-7) antagonist). The representative immunoblots of protein expressions (UCP1, CITED1, aP2 and actin) and the statistics (mean ± SEM, n = 3, from 3 independenet experiments; normalized to actin densities) are shown accordingly. **p* < 0.05, *vs.* control.

**
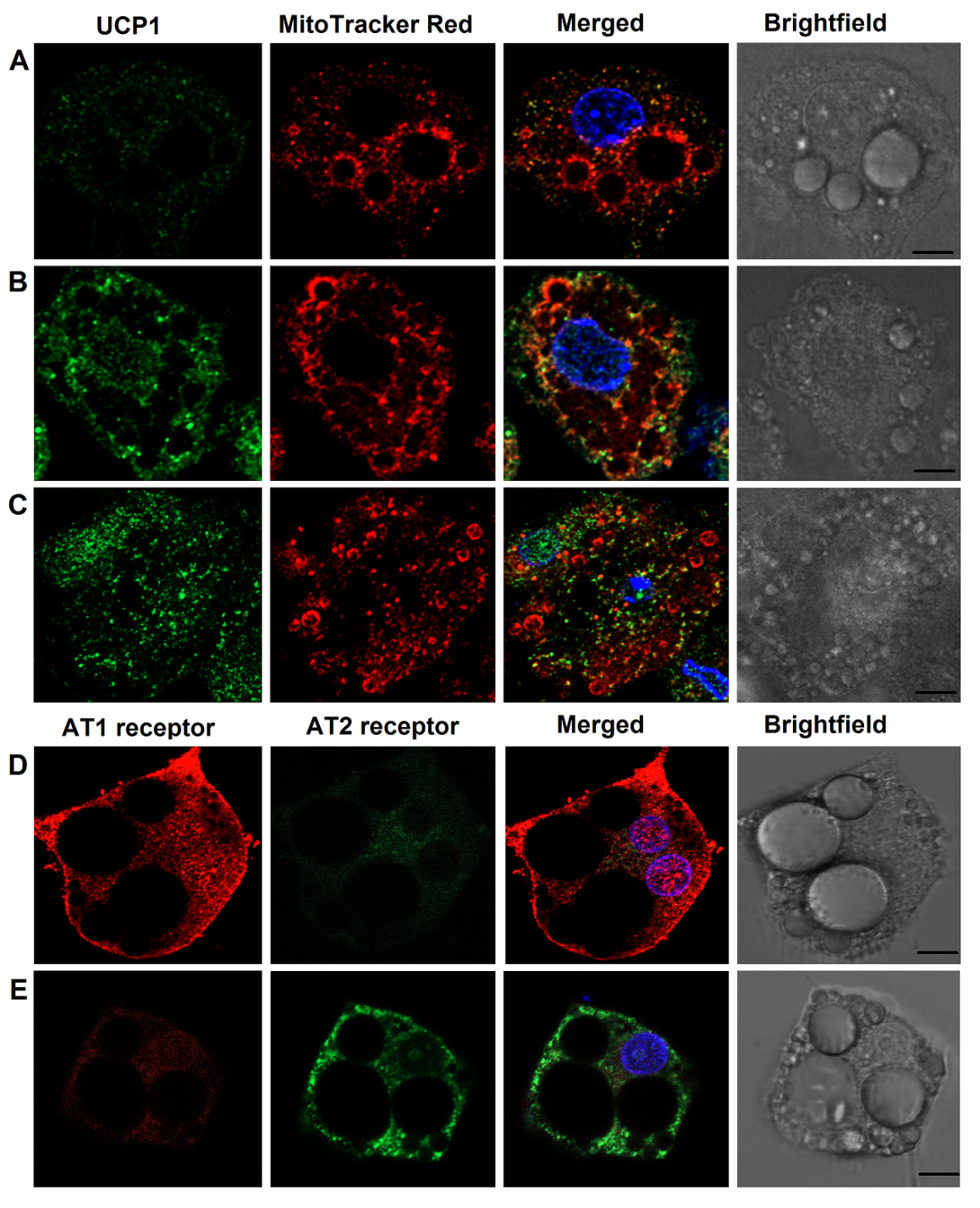
**

**Supplemental Figure 2S: [A - C]** The representative confocal images (from 3 separate experiments) of immunostained UCP1 (rabbit UCP1-IgG with bovine anti-rabbit IgG-CFL488), mitochondria (detected by MitoTracker Red) and their corresponding merged and bright-field images in [A] mouse white adipocytes (White adipocytes), [B] mouse white adipocytes treated for 4 - 5 days with AngII plus ZD7155 (AT2R activation) (Beige adipocytes) or mouse brown adipocytes (Classical brown adipocytes). **[D and E]** The representative confocal images (from 3 separate experiments) of immunostained AT1R (goat AT1R-IgG with donkey anti-goat IgG-CFL594), AT2R (rabbit AT2R-IgG with bovine anti-rabbit IgG-CFL488) and their corresponding merged and bright-field images in [D] mouse white adipocytes transfected with AT2R-siRNA for 2 days, or [E] mouse white adipocytes transfected with AT1R-siRNA for 2 days. Hoechst 33342 (NucBlue Live ReadyProbes Reagent) was used to stain the nuclei. Scale bars = 10 µm.

**
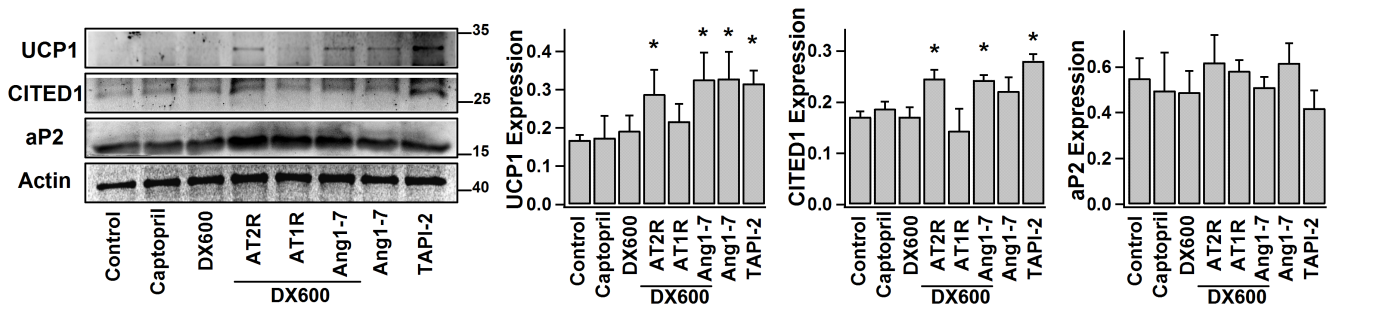
**

**Supplemental Figure 3S:** Mouse white adipocytes (day8) were treated for 4 days, without (-) (control) or with (+) 1 µM captopril (ACE inhibitor), 1 µM DX600 (a potent ACE2 inhibitor), AngII plus ZD7155 (AT2R activation), AngII plus PD123319 (AT1R activation), 100 nM Ang(1-7) or 10 µM TAPI-2 (inhibitor of TACE/ADAM17). The representative immunoblots of protein expressions (UCP1, CITED1, aP2 and actin) and the statistics (mean ± SEM, n = 3, from 3 independent experiments; normalized to actin densities) are shown accordingly. **p* < 0.05, *vs*. control.


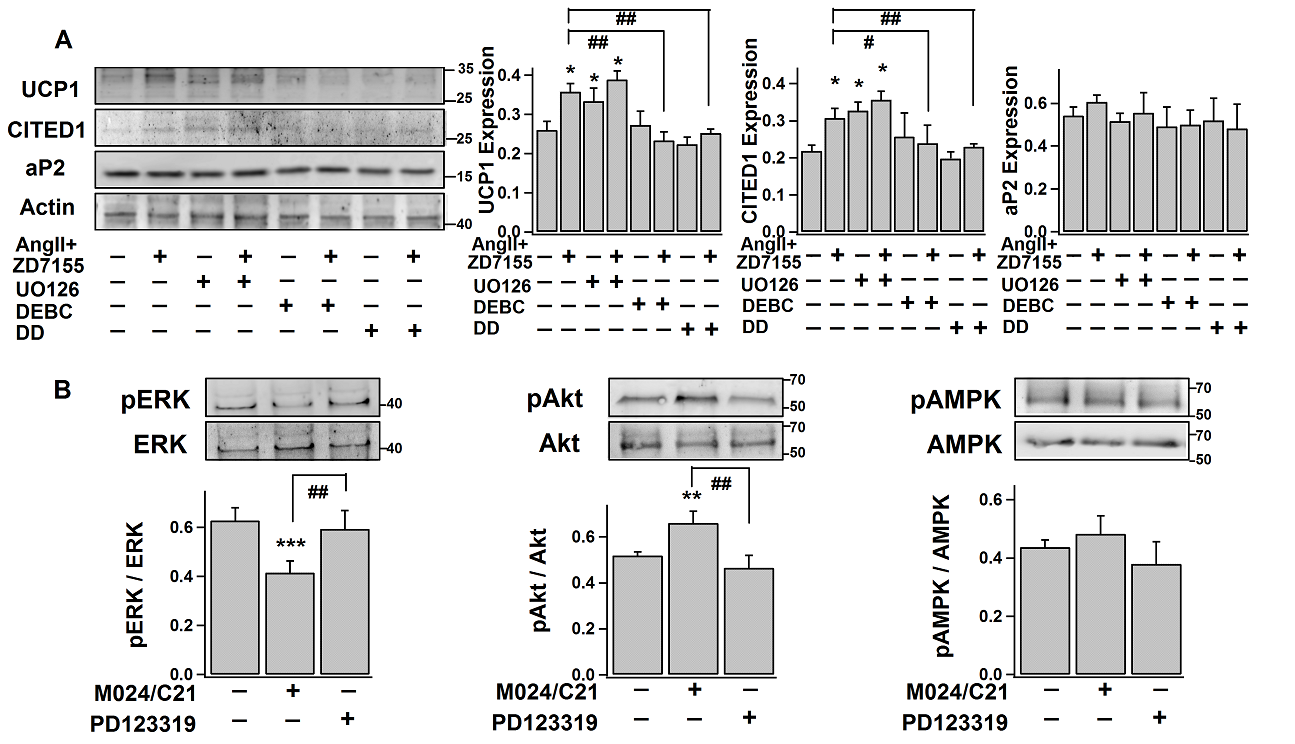


**Supplemental Figure 4S:** **[A]** Mouse white adipocytes (day8) were maintained in the presence (+) or absence of (-) AngII plus ZD7155 (AT2R activation) for 4 days. To inhibit ERK1/2, Akt or AMPK signaling, cells were pre-treated for 60 min with (+) or without (-) 10 µM UO126 (selective inhibitor of MEK1/2), 10 µM 10-DEBC (selective inhibitor of Akt/PKB), or 10 µM dorsomorphin (DD, selective inhibitor of AMPK), respectively. The representative immunoblots of protein expressions (UCP1, CITED1, aP2 and actin) and the statistics (mean ± SEM, n = 3, from 3 independent experiments; normalized to actin densities) are shown accordingly. **[B]** Mouse white adipocytes (day8) were treated without (-) or with (+) 100 nM M024/C21 (AT2R activation) or 1 µM PD123319 (AT2R antagonist) for 30 mins. Top panels, representative immunoblots; bottom panels, statistics (mean ± SEM, n = 3, from 3separate experiments) of the optical density ratio between pERK and ERK, pAkt and Akt, or pAMPK and AMPK. **p* < 0.05, ***p* < 0.01, ****p* < 0.001 vs. control; # *p* < 0.05 and ## *p* < 0.01 between indicated pairs.


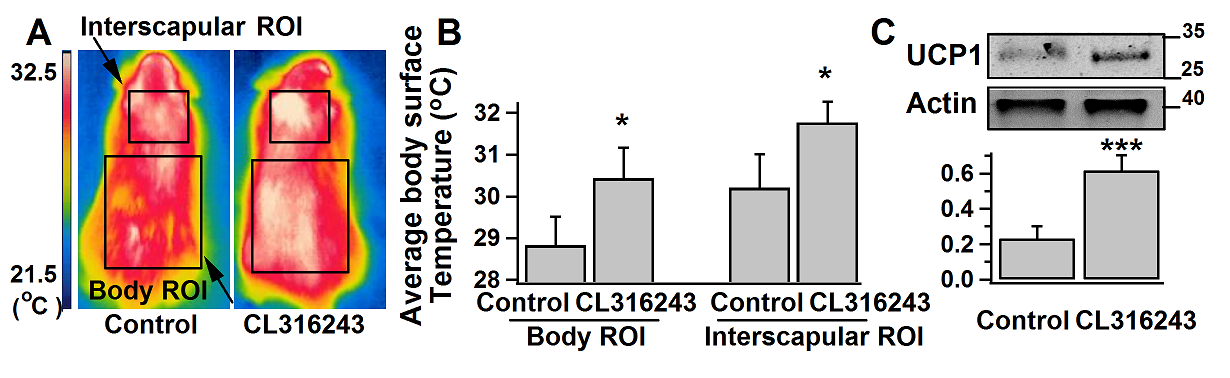


**Supplemental Figure 5S:** Mice (C57BL/6J, 8 - 10 weeks old, male) were subjected to intraperitoneal injection of CL316,243 (0.5 mg/kg/day, dissolved in 100 µl of PBS) for 5 days. [A and B] The representative thermographic images and the statistical analyses of the average surface temperature within a region of interest (ROI), at body or interscapular area are shown accordingly (n = 4 mice per group). The representative immunoblots of protein expression in IgWAT isolated from differently treated mice, and the statistics (n = 4 mice per group; normalized to actin densities) are shown accordingly. **p* < 0.05, *** *p*<0.001 vs. control.


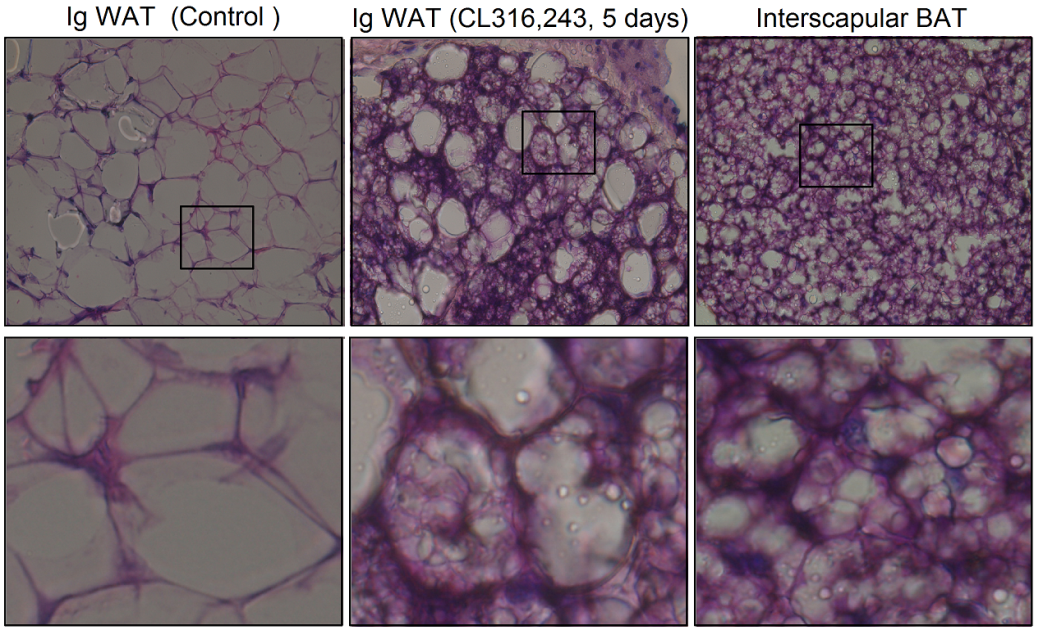


**Supplemental Figure 6S:** The representative hematoxylin and eosin staining images (from 3 mice per group) of IgWAT of control mice (no treatment), IgWAT of mice treated with CL316,243 (0.5 mg/kg/day, for 5 days), and interscapular BAT of control mice (no treatment) (upper lane- 400x). White adipocytes, Beige adipocytes, and Classical brown adipocytes are shown in the lower lane.

**
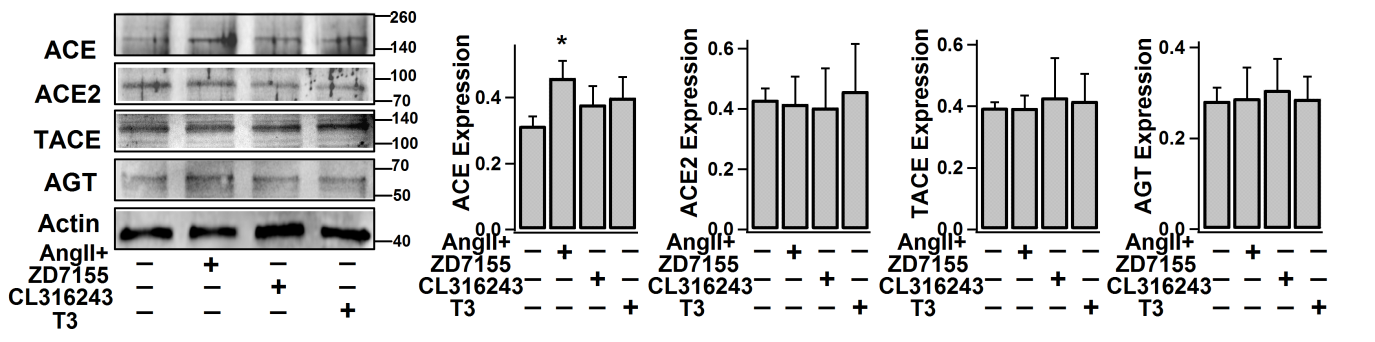
**

**Supplemental Figure 7S:** Human white adipocytes (day14) were treated without (-) (control) or with (+) AngII plus ZD7155 (AT2R activation), 1 µM CL316243 (a selective β3-adrenoceptor agonist) or 50 nM triiodo-L-thyronine (thyroid hormone T3) for 4 - 5 days. The representative immunoblots of protein expressions (ACE, ~ 195 kDa; ACE2, ~ 97 kDa; TACE, ~ 120 kDa; AGT, ~ 60 kDa; actin) and the statistics (mean ± SEM, n = 4, from 4 independenet experiments; normalized to actin densities) are shown accordingly. **p* < 0.05, *vs*. control.
